# SARS-CoV-2: una nueva amenaza

**DOI:** 10.1515/almed-2020-0045

**Published:** 2020-09-03

**Authors:** Cristina A. López Rodríguez, Marc Boigues Pons, Bibiana Quirant Sánchez, Aina Teniente Serra, Joan Climent Martí, Eva Ma Martínez Cáceres

**Affiliations:** Servicio de Inmunología, Laboratori Clínic Metropolitana Nord, Hospital Universitario Germans Trias i Pujol, Badalona, Barcelona, Spain

**Keywords:** COVID-19, pandemia, respuesta inmunitaria, SARS-CoV-2

## Abstract

**Objetivos:**

Aportar una breve revisión del conocimiento actual sobre el virus SARS-CoV-2, cuya irrupción ha causado un gran impacto en la sociedad a escala mundial.

**Contenido:**

Esta revisión aporta una visión general de diversos aspectos del virus y de la respuesta inmunitaria que desencadena, así como aproximaciones diagnósticas y terapéuticas que se están llevando a cabo.

**Resumen:**

SARS-CoV-2 es un virus RNA con características peculiares que lo diferencian de sus predecesores SARS-CoV y MERS. Dada sus características estructurales y su patogenia, es capaz de provocar diversas manifestaciones clínicas según avanza la enfermedad. Se ha demostrado que el sistema inmunitario juega un papel importante en la respuesta frente a este virus y, por ende, es crucial el estudio de los anticuerpos y las poblaciones linfocitarias durante los distintos estadios de la enfermedad.

**Perspectiva:**

El conocimiento del efecto del virus y la respuesta inmunitaria es determinante para el desarrollo de vacunas, terapias y técnicas diagnósticas de calidad, esenciales para el control y la erradicación de la enfermedad.

## Introducción

### SARS-CoV-2

A finales del mes de Diciembre del 2019, se reportaron nuevos casos de neumonía de causa desconocida cuyo foco se encontraba en un mercado mayorista de mariscos y animales en Wuhan, provincia de Hubei, China [1].Tras el repunte de estos casos, se empezó a analizar muestras del líquido broncoalveolar (BAL) de pacientes con neumonía y síntomas compatibles, con el fin de estudiar el material genético y obtener la secuenciación genómica del agente infeccioso. Finalmente se detectó un nuevo coronavirus, *SARS-CoV-2* siendo identificado como β-coronavirus, con características similares pero distinto de otros virus causantes de epidemias como el SARS y el MERS [1].

### Características del nuevo SARS-CoV-2

Los Coronavirus (CoV) pertenecen a la familia *Coronaviridae,* los cuales son virus RNA envueltos, monocatenarios de 26 a 32 kilobases de longitud [2].

Estos virus pueden causar una amplia gama de enfermedades tanto respiratorias como entéricas, hepáticas y neurológicas. Los coronavirus se dividen en cuatro subfamilias: α, β, γ y δ-CoV. Estas infecciones humanas por CoV son causadas por α y β-CoV. Otros β-CoV, como son el SARS-CoV y MERS-CoV, conducen a infecciones respiratorias graves y potencialmente mortales [3].

La estructura del SARS-CoV-2 es similar a los otros β-CoVs: El virión presenta una nucleocápside constituida por ARN y la proteína fosforilada de la nucleocápside (N). Esta nucleocápside se encuentra recubierta de una doble capa de fosfolípidos donde se encuentran dos proteínas “espiga”: la glicoproteína trimérica de espiga (S) y la hemaglutinina esterasa (HE) [3]. Ésta última sirve como una enzima destructora de receptores que facilitará la liberación del virión de las células infectadas y permitirá escapar de la unión a las células huésped no permisivas [4]. La proteína S es una proteína de fusión viral que promueve la unión de las membranas viral y celular durante la entrada. Ésta también suele ser el antígeno principal de los anticuerpos neutralizantes generados durante la infección y uno de los focos del diseño de vacunas [4].

**Figura 1:**
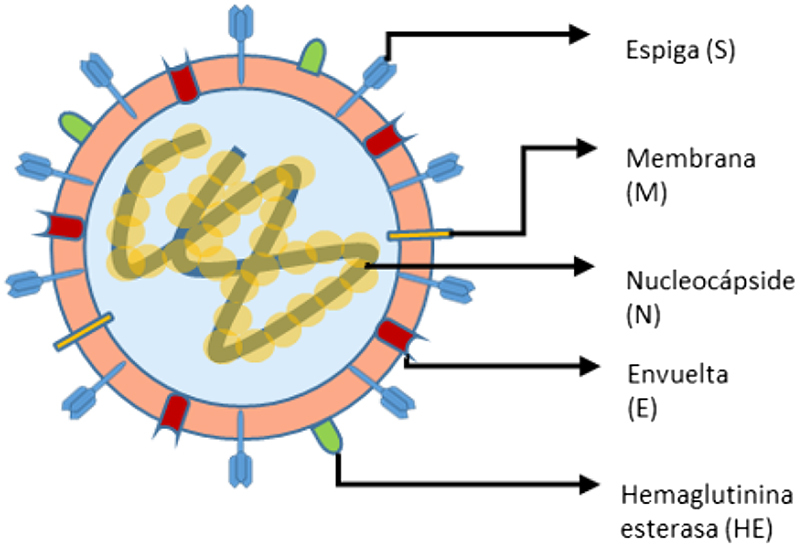
Estructura del SARS-CoV-2. Adaptado de Jin et al. [3].

A lo largo de la cubierta, también se encuentran las proteínas de membrana (M) y envuelta (E) [3] (Figura 1).

### Patogénesis

El SARS-CoV-2 utiliza el receptor ACE2 (enzima convertidora de angiotensina 2) para su entrada en el huésped, al igual que SARS-CoV [5]. Este tipo de receptor se encuentra localizado en los pulmones, corazón, riñones e intestino [3] y mayoritariamente asociado a enfermedades cardiovasculares como la hipertensión, donde se ha observado una mayor expresión de éstos por mecanismo compensatorio de la propia enfermedad [6].

El virión se une al receptor ACE2 mediante la glicoproteína S. La glicoproteína S incluye dos subunidades, S1 y S2. La subunidad S1 determina el tropismo celular mediante el dominio de unión al receptor (RBD) mientras que la S2 media la fusión de la membrana celular del virus gracias a dos dominios en tándem (HR1 y HR2) [7].

Después de la unión al receptor, se produce un cambio conformacional en la proteína S, facilitando la fusión de la envoltura viral con la membrana celular a través de la vía endosómica. A continuación, el SARS-CoV-2 libera ARN en la célula huésped. El ARN del genoma se traduce en poliproteínas de replicasa viral pp1a y 1ab, que a su vez se dividen en pequeños productos mediante proteinasas virales. La polimerasa produce una serie de ARNm por transcripción discontinua y finalmente se traduce en proteínas virales. Las proteínas virales y el ARN del genoma se ensamblan en viriones en el retículo endoplásmico y en el aparato de Golgi, se transportan a través de vesículas y se liberan de la célula [8].

### Etapas de la infección y características clínicas:

En varios estudios como el de *Siddiqi HK* et al., se proponen tres estadios clínicos de la enfermedad [9]

**Etapa I**: Establecimiento temprano de la enfermedad.

Esto implica un período de incubación de entre 1 a 14 días, principalmente de 3 a 7 días, asociado con síntomas leves y a menudo no específicos, como malestar general, fiebre y tos seca [7]. En esta etapa, el SARS-CoV-2 se multiplica y establece su residencia en el huésped, centrándose principalmente en el sistema respiratorio. Durante la etapa de incubación el paciente es contagioso y el virus puede transmitirse a través de gotículas, secreciones respiratorias y contacto directo [7]. Las alteraciones de laboratorio más frecuentes en esta fase son la elevación de la proteína C reactiva (PCR), enzimas hepáticos y ligeras linfopenia y neutrofilia [9].

**Etapa II (moderada)**: compromiso pulmonar (IIa) sin y (IIb) con hipoxia.

Durante la segunda semana, hasta un 80% de pacientes pueden desarrollar una neumonía viral, con tos, disnea, fiebre y posiblemente hipoxia (definido como un PaO_2_ / FiO_2_ de <300 mmHg). A nivel radiológico, se observan infiltrados bilaterales u opacidades en *vidrio esmerilado* tanto en las imágenes con radiografía de tórax como en tomografía axial computarizada. Los análisis de sangre revelan linfopenia, junto con transaminitis. Los marcadores de inflamación sistémica (IL-6, velocidad de sedimentación globular (VSG), PCR, dímero D y ferritina) se encuentran elevados, pero no excesivamente [9].

**Etapa III (severa)**: Hiperinflamación sistémica.

Esta es la etapa más grave de la enfermedad a la que llega aproximadamente un 15% de pacientes y que se manifiesta como un síndrome de hiperinflamación sistémica extrapulmonar. En esta etapa, los marcadores de inflamación sistémica (entre ellos la IL-6) están muy elevados e incluso se puede llegar a desarrollar una forma similar a la linfohistiocitosis hemofagocítica (SHLH). Los pacientes con enfermedad más grave también presentan una elevación de citocinas proinflamatorias, dímero D, ferritina, troponinas y prohormona N-terminal del péptido natriurético cerebral (NT-proBNP) además de una marcada neutrofilia y linfopenia. También puede desarrollar shock, vasoplejía, insuficiencia respiratoria e incluso colapso cardiopulmonar. Por último, puede llegar a dar un fallo multiorgánico [9].

## Respuesta inmunitaria

### Respuesta celular

La respuesta inmunitaria en los pacientes con COVID-19 se puede dividir en tres fases, las cuales se solapan con las etapas clínicas anteriormente descritas:

(1) Fase de viremia. En esta fase la presencia del ARN viral en sangre aumenta exponencialmente durante la primera semana. La respuesta inicial de tipo innato, con producción de interferones tipo-I (IFN)-I en las vías aéreas es clave para el control de la replicación viral y la puesta en marcha de una correcta respuesta celular inmunitaria efectiva [10].
(2) Fase aguda o de neumonía. Tiene lugar a los 7-10 días del inicio de la infección. Se caracteriza por un estado inflamatorio con neutrofilia, aumento de citocinas proinflamatorias y quimiocinas (interleucina (IL)-6, factor de necrosis tumoral (TNF)-α, IFN-I y -II, IL-8, CXCL-10), disminución de linfocitos T y B en sangre periférica y alteración de diversos parámetros bioquímicos y hematológicos, cómo incremento de dímero D, ferritina, lactato deshidrogenasa (LDH), PCR y disminución de la albúmina 56 [11]. Durante esta etapa la carga viral ha alcanzado su nivel máximo y comienza a disminuir. Llegados a este punto, el paciente puede evolucionar de manera diferente en función de la capacidad de su sistema inmunitario de controlar la infección viral.
(3A) Fase de recuperación. Si la respuesta inmunitaria del paciente es suficiente para hacer frente a la infección y la carga viral disminuye paulatinamente, los marcadores de inflamación se reducen y las poblaciones linfocitarias se recuperan [12].
(3B) Fase severa. En caso de que el sistema inmunitario no sea capaz de controlar la infección, el número de linfocitos disminuye aún más mientras que la carga viral y los marcadores de inflamación siguen aumentado. Los pacientes en este estadio empeoran progresivamente pudiendo llegar a morir [12].

Aunque los mecanismos subyacentes a este comportamiento inflamatorio del sistema inmunitario son poco conocidos, se han identificado algunos procesos celulares que nos pueden ayudar a comprender la evolución de la enfermedad.

Por lo que se refiere a la respuesta inicial frente al virus, una expresión de IFN-I poco potente o retrasada en el tiempo se ha asociado a un peor pronóstico de la enfermedad [10]. En este sentido, se ha descrito que el propio virus es capaz de disminuir la expresión de genes de IFN-α e IFN-β en monocitos durante la infección [13]. Esto implica una reducción de la respuesta antiviral asociada a estas moléculas y de la expresión de otras moléculas como las del complejo mayor de histocompatibilidad (MHC) de clase I, catepsinas, proteínas lisosomales y del proteasoma, todas ellas implicadas en la presentación de antígenos y la respuesta inmunitaria de tipo 1 (antiviral) [13], [14].

Algunos pacientes COVID-19 presentan una liberación masiva de citocinas llamada “Tormenta de citocinas”, proceso que se asemeja al que ocurre en otras patologías como el SHLH. Las causas exactas que inducen esta liberación no se conocen, aunque se postula que el bloqueo primario de la respuesta de IFN-I puede producir de alguna manera una respuesta secundaria exagerada de otras citocinas [15]. Las citocinas que se pueden observar elevadas son la IL-6, el TNF-α, el IFN-γ o la IL-8, entre otras [15], [16]. La IL-1β, una de las principales citocinas pro-inflamatorias, se encuentra en niveles normales en la sangre periférica de estos pacientes, ya que ésta tiene una semivida corta y suele degradarse rápidamente. Este aspecto se tiene que tener en cuenta a la hora de monitorizar los pacientes con tratamientos biológicos, dado que la IL-1β no es un buen parámetro para monitorizar [17].

Por lo que respecta a las subpoblaciones linfocitarias, en los pocos estudios realizados hasta el momento los linfocitos T CD4^+^ y CD8^+^ son las poblaciones que presentan una mayor disminución en cuanto a número, seguidas por los linfocitos NK y B. Este descenso parece acentuarse en los pacientes más graves y en aquellos con mayores niveles de citocinas proinflamatorias. También se ha observado una disminución en los linfocitos T reguladores [18].

Una vez llegados a la fase aguda, los pacientes que empiezan a controlar el virus son aquellos que consiguen desarrollar una respuesta inmunitaria específica adecuada, tanto humoral como celular [19]. En este sentido, se han observado incrementos en sangre periférica de los porcentajes de plasmablastos, productores de anticuerpos, (CD19^+^CD27^high^CD38^high^) y linfocitos T colaboradores foliculares *(T folicular helper -Tfh-)* circulantes (CD4^+^CXCR5^+^ICOS^+^PD-1^+^) a partir del día 7 de desarrollar la clínica [20]. También se ha visto un incremento de linfocitos T CD8^+^ con fenotipo activado (CD38^+^HLA-DR^+^) en pacientes con una buena evolución y en aquellos que responden a los tratamientos inmunomoduladores [21]. Este aumento no se aprecia en los pacientes más graves, los cuales además presentan mayor cantidad de linfocitos T CD8^+^ con fenotipo exhausto (PD-1^+^ CTLA-4^+^ TIGIT^+^) [22].

La respuesta celular que se desarrolla es fundamentalmente de tipo 1. Estudios en pacientes infectados por el SARS-CoV han demostrado la presencia de linfocitos T CD4^+^ y CD8^+^ productores de IFN-γ específicos frente a diversos antígenos virales [23]. Se han descrito poblaciones T CD4^+^ de memoria frente al SARS-CoV residentes en las vías aéreas incluso 11 años después de la infección [24]. Estas poblaciones celulares son capaces de reaccionar frente al virus rápidamente, liberando IFN-γ, promoviendo el desplazamiento de células dendríticas pulmonares cargadas con el antígeno a los nodos linfáticos mediastínicos y atrayendo linfocitos T CD8^+^ específicos frente al virus al pulmón [23], [24], [25]. Hacen falta más estudios en sangre periférica de subpoblaciones linfocitarias minoritarias que permitan monitorizar estos cambios durante la evolución de la enfermedad.

### Respuesta humoral

Al tratarse de una enfermedad producida por un nuevo virus, existe poco conocimiento acerca de la respuesta humoral que se produce en los pacientes infectados. En este contexto, es importante conocer tanto la cinética de seroconversión, los títulos de los anticuerpos, la duración en el plasma de los pacientes una vez superada la infección y su capacidad como anticuerpos neutralizantes.

Por ello, algunos autores se han centrado en la respuesta humoral de otros virus con los que el SARS-CoV-2 presenta cierta similitud, como el SARS-CoV. En el estudio realizado por Li et al. [26], analizaron la respuesta inmune humoral de 20 pacientes en donde pudieron observar la cinética de aparición de las inmunoglobulinas desde la semana de inicio de la enfermedad hasta la semana 12 [26]. Destacaron la positividad de anticuerpos IgG a partir de la semana 3, pudiéndose mantener hasta 3 meses tras los primeros síntomas. Estos anticuerpos reconocían mayoritariamente a las proteínas S y N del virus [27]. Por otro lado, los anticuerpos tipo IgM aparecían en los primeros días y desaparecían progresivamente hasta la semana 12. Ante estos resultados, los autores sugieren que los anticuerpos IgG contra el SARS-CoV pueden jugar un papel importante en la protección frente a la infección por SARS [27].

Más recientemente estudios con el nuevo SARS-CoV-2 han podido demostrar una cinética similar, a pesar de que no en todos ellos se analizan los mismos antígenos o el mismo tipo de pacientes.

**Figura 2:**
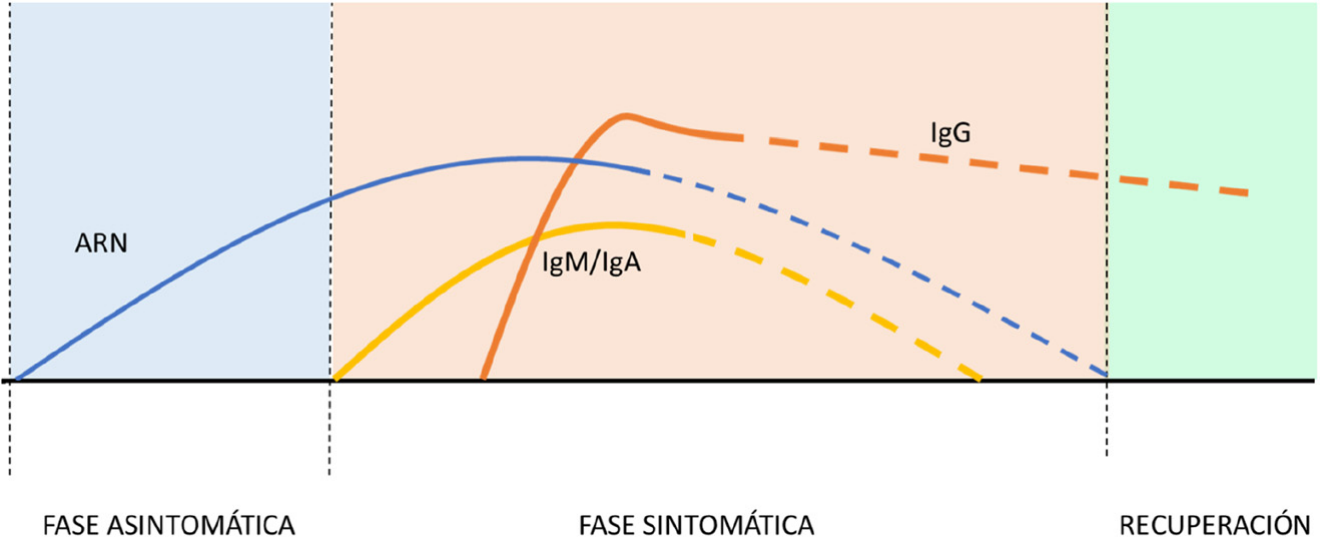
Representación de la cinética de ARN y anticuerpos IgG, IgA e IgM anti SARS-CoV-2 en las diferentes fases clínicas de la enfermedad COVID19.

Guo et al. [28] detectaron la aparición de anticuerpos IgM e IgA a los 5 días del inicio de los síntomas, pudiéndose detectar hasta 3 semanas más tarde. Por otro lado, la presencia de IgG se detectó alrededor de los 14 días después del inicio de la clínica, aunque algunos autores apuntan a que la IgG podría detectarse antes, alcanzando su *plateau* a los 21 días, existiendo variación según paciente [29], [28]. [Figura 2]

Estos resultados son similares a los reportados por otros autores como Theravajan et al. que analizaron la cinética de la respuesta inmunológica en un paciente australiano con SARS-CoV-2 [20].

Estudios como el de Zhao et al. [30], observaron una cinética de aparición de anticuerpos totales, IgM e IgG a 11, 12 y 14 días respectivamente, alcanzando una seropositividad del 50% en el día 11 y un 100% a día 39 [30].

La aparición de títulos elevados de IgG antes de los 14 días se ha relacionado con la severidad de la enfermedad [30], [31]. La causa exacta de este fenómeno se desconoce actualmente, aunque se cree que niveles mayores de anticuerpos conducen a un incremento de la formación de inmunocomplejos fijadores del complemento en los tejidos pulmonares, con la consiguiente inflamación y daño tisular [32]. También se sospecha de la existencia de anticuerpos relacionados con el fenómeno ADE (*antibody-dependent enhancement*), los cuales se han observado en el SARS-CoV. Este fenómeno se caracteriza por la unión de los anticuerpos IgG al virus [33], [34]. El inmunocomplejo virus-IgG se uniría por la fracción constante (Fc) de las IgG a los receptores *Fc gamma receptor* (FcγR) presentes en la superficie de monocitos, macrófagos y linfocitos B. La unión Fc- FcγR facilitaría la entrada del virus en estas células. Esta infección de las células no daría lugar a una replicación viral funcional pero induciría la activación de las células infectadas y la generación de citocinas pro-inflamatorias como el TNF-α y la IL-6 incrementando la inflamación de los pacientes [34].

## Técnicas de diagnóstico en el laboratorio

Con la nueva aparición de casos diagnosticados de COVID-19, empezaron a desarrollarse técnicas diagnósticas en el laboratorio para su detección precoz y posible relación con el estadio de inmunización de los pacientes infectados.

Actualmente, se disponen de diversas técnicas para el diagnóstico de laboratorio del COVID-19:

1) Técnicas de detección de ácidos nucleicos: Esta técnica se basa en la llamada RT-PCR, o reacción en cadena de la polimerasa con transcriptasa inversa, que consiste en la amplificación del genoma del virus y es actualmente la técnica *Gold Standard.* Para esta técnica, se recomienda recoger muestras tanto del tracto respiratorio superior como inferior para una mayor sensibilidad en la prueba [35]. La ventaja es la alta especificidad y sensibilidad que presenta (sobre todo dentro de los 7 primeros días del inicio de los síntomas) y en el procesamiento de gran número de muestras a la vez. Es cierto que en algunos casos puede dar falsos negativos [36], [37] debido a errores preanalíticos (recogida inadecuada de la muestra, mala conservación) [38] o por baja carga viral en la muestra a testar [39]. La determinación de la presencia del virus por RT-PCR puede perder sensibilidad a lo largo del tiempo, llegando a alcanzar una sensibilidad menor al 50% después del día 14 de inicio de los síntomas. Por ello, se han propuesto técnicas complementarias como la determinación de los anticuerpos para clasificar a estos pacientes [28].
2) Técnicas de detección de antígeno: Se basa en la detección de antígenos propios del virus (normalmente la proteína S o la N) pero no existen muchos estudios acerca de la sensibilidad y especificidad que ofrecen este tipo de pruebas. En el estudio llevado a cabo por Diao et al., [40] mostraron que en muestras de pacientes que habían sido positivas por RT-PCR con Ct (Ciclo umbral) <40, la sensibilidad era del 68% y la especificidad del 100%, mientras que con Ct < 30, la sensibilidad era del 98% y especificidad del 100% [40]. La principal ventaja es el tiempo de procesamiento, ya que se trata de ensayos de inmunocromatografía (test rápidos o llamados *Point Of Care*) cuyo resultado se obtiene en unos 15-30 minutos. No obstante, su utilidad en la práctica diaria es aún controvertida.
3) Técnicas de detección de anticuerpos: Consiste en la detección de anticuerpos tipo IgA, IgM o IgG en pacientes infectados por COVID-19. Existen técnicas semicuantitativas (ensayo tipo ELISA o CLIA) o cualitativas (inmunocromatografía, *Point of Care*) para su detección. Dado que la respuesta inmune es detectable principalmente entre 7 y 11 días después de la exposición al virus aunque algunos pacientes pueden desarrollar anticuerpos antes [38], esta prueba no debe realizarse la primera semana desde el inicio de síntomas por baja sensibilidad. Sin embargo, a partir del día 7, la determinación conjunta de RT-PCR y anticuerpos puede incrementar la sensibilidad diagnóstica hasta un 95% [28]. A partir del día 7 de inicio de los síntomas, la sensibilidad y especificidad que pueden alcanzar estas pruebas mediante inmunocromatografía, pueden llegar a ser de un 88% y 90,6% respectivamente [41]. Los resultados de los estudios realizados mediante ELISA, fueron muy similares [42]. No obstante, al igual que con las técnicas de detección de antígeno, son necesarios más estudios para determinar la sensibilidad y especificidad de las diferentes técnicas [42].

Por otro lado, se ha observado que la combinación de RT-PCR y detección de anticuerpos específicos incrementa el porcentaje de detección hasta el 100% a partir de los 15 días del inicio de los síntomas [28], [29].

Las ventajas y desventajas de las técnicas disponibles en el laboratorio se resumen en la Tabla 1.

**Tabla 1:** Ventajas y desventajas de las técnicas disponibles en el laboratorio para el diagnóstico de infección por SARS-CoV-2.

	Ventajas	Desventajas
RT-PCR	Elevado número de procesamiento de muestras Elevada especificidad Elevada sensibilidad los primeros 7 días tras el inicio de síntomas	Dificultad en la recogida de muestra (nasofaringe) Tiempo de procesamiento largo
Antigeno viral	Rápido	Detección de pocas muestras a la vez Pocos estudios de sensibilidad y especificidad Se requiere mínimo de carga antigénica detectable
Anticuerpos	Rápido Buena sensibilidad a partir del día 7 post-inicio de sintomatologia Elevada especificidad A partir del día 7, los anticuerpos junto a la RT-PCR aportan una elevada sensibilidad diagnóstica Muestras a testar (suero, plasma y sangre periférica)	Cinéticas de IgA, IgM e IgG variables entre diferentes pacientes

### Importancia de la detección de anticuerpos

La determinación de anticuerpos puede ser de utilidad para la confirmación del diagnóstico de la infección, de manera complementaria a otras herramientas como la RT-PCR , disminuyendo el porcentaje de falsos negativos y, consecuentemente, aumentando la sensibilidad diagnóstica [28]. Además ayudan a evaluar el estadio en el que se encuentra el paciente (Tabla 2) [44].

**Tabla 2:** Interpretación de resultados de RT-PCR junto a anticuerpos IgA/IgM e IgG en infección por SARS-CoV-2. Adaptado de [43].

Resultado			Significado clínico
PCR	IgA/IgM	IgG	
−	−	−	Negativo
+	−	−	Periodo ventana
+	+	−	Fase temprana de la infección
+	+	+	Fase activa de la infección
+	−	+	Fase final de la infección
−	+	−	Fase temprana con falso negativo. Realizar PCR de confirmación
−	+	+	Enfermedad en evolución. Realizar PCR de confirmación
−	−	+	Infección pasada y curada

Por tanto, teniendo en cuenta lo dicho anteriormente y atendiendo al documento propuesto por la *Sociedad Española de Inmunología* [44], la detección de Ac anti-SARS-CoV-2 también serían de utilidad en pacientes con clínica compatible con COVID-19 y RT-PCR negativa.

No existe mucha información sobre la inmunidad protectora que se puede desarrollar frente al SARS-CoV-2. Actualmente, sólo existe un estudio que analiza esta inmunidad en el mono *Rhesus* [45]. En este trabajo, se observó que los monos que son capaces de generar un título elevado de anticuerpos neutralizantes en una etapa temprana de la infección, posteriormente no se reinfectan con el virus. Por tanto, aunque hacen falta más estudios, estos resultados apoyan la gran utilidad de la determinación de anticuerpos a nivel de la población general para detectar los individuos que hayan pasado la infección de forma asintomática, así como aquellos que hayan podido generar inmunidad protectora [44].

### Reactivos comerciales para la detección de antígenos y anticuerpos

Actualmente, se dispone de una gran variedad de reactivos comerciales, muchos en periodo de validación y otros con certificado CE (Conformidad Europea), para la detección tanto de antígenos como de anticuerpos anti-SARS-CoV-2 por diferentes técnicas. Éstos se han descrito y comparado de manera exhaustiva en el repositorio “*Foundation for Innovative New Diagnostics (FIND)*” [46].

No obstante, es recomendable realizar validaciones antes de su implantación a nivel rutinario en los laboratorios. Además, debido a la gran demanda durante la pandemia, a nivel práctico, antes de seleccionar un reactivo debe considerarse su disponibilidad y periodo de entrega.

En un estudio reciente, dirigido por *Lassaunière* et al., se analizaron diferentes test serológicos (dos ELISA y seis inmunocromatografías *Point of Care*) de distintas empresas con diferentes especificidades y sensibilidades y se realizó una comparativa entre los métodos utilizados y sus validaciones internas, facilitando así la elección del test serológico [47]. Así mismo, en Estados Unidos se ha iniciado el *“COVID-19 testing project”,* un proyecto para evaluar y comparar las características de los reactivos que existen en el mercado [48]

También se están desarrollando inmunoensayos de quimioluminiscencia (CLIA) en plataformas automatizadas. Existen pocos estudios que utilicen esta técnica como test diagnóstico, pero autores como Lin et al. [49], en una comparativa entre la técnica ELISA y CLIA, observaron una mayor sensibilidad y especificidad en el ensayo de quimioluminiscencia.

Aunque COVID-19 es una nueva enfermedad, es importante la validación previa a su utilización para poder valorar posibles reacciones cruzadas con otros coronavirus y obtener un resultado óptimo en el diagnóstico y seguimiento de la enfermedad.

## Tratamiento de COVID-19

Actualmente, apenas dos meses tras el inicio de la pandemia en nuestro país, no existe una aproximación terapéutica establecida. Existe gran número de ensayos clínicos que intentan identificar/reposicionar fármacos para tratar el COVID-19, por lo que el abanico terapéutico cambia continuamente.

Los fármacos utilizados en el tratamiento de los pacientes COVID-19 se pueden dividir en dos grandes grupos: los que intentan frenar la replicación viral y aquellos que tienen como objetivo reducir la hiper-inflamación sistémica [50].

Entre los fármacos que bloquean la replicación viral más estudiados podemos encontrar:

Lopinavir/ritonavir. El lopinavir es un inhibidor de la proteasa del VIH que ha sido utilizado ampliamente por las autoridades chinas en el tratamiento de pacientes COVID-19 [51].
Remdesivir. Se trata de un análogo nucleotídico que interfiere en la polimerización del ARN viral. Aunque hay estudios que asocian este fármaco a un beneficio clínico aún se requiere de una mayor evidencia científica para su uso generalizado en pacientes COVID-19 [52]
Hidroxicloroquina/cloroquina. Estos fármacos antipalúdicos han demostrado tener cierta capacidad antiviral in vitro frente al SARS-CoV-2. Se ha observado que interfieren tanto en la entrada como en la replicación del virus. También pueden interferir en la señalización de los TLR (*toll like receptor*s), reduciendo así la activación inmunitaria [53]. A pesar de estas características, su beneficio clínico es aún controvertido [54].

En relación con los fármacos utilizados para reducir la hiper-inflamación, en esta enfermedad, hay que tener en cuenta cómo funciona la respuesta inmunitaria inicial a cualquier patógeno. En ella los macrófagos, granulocitos, células dendríticas y las células epiteliales y endoteliales son capaces de detectar la presencia de proteínas y ARN/ADN del patógeno a través de receptores tipo TLR y NLR (NOD like receptors), entre otros receptores. La activación de estos receptores induce la liberación de IL-1β e IL-6. Estas citocinas a su vez inician una cascada de inflamación que implica la producción de otras citocinas y quimiocinas, la inducción de fiebre, la producción de reactantes de fase aguda en el hígado y la generación de leucocitos en la médula ósea [55], [56].

Así, dentro del grupo de fármacos cuyo objetivo es reducir la hiper-inflamación se han utilizado diferentes tratamientos, con distinta especificidad en cuanto a su mecanismo de acción:

Corticoides. Han sido ampliamente utilizados en pacientes con COVID-19. Sin embargo, su utilidad no ha sido claramente demostrada. Existen varios estudios que indican que estos fármacos no sólo no son beneficiosos, sino que incluso pueden ser perjudiciales, aunque existen datos controvertidos [54], [57]. Actualmente la Agencia Española del Medicamento y Productos Sanitarios (AEMPS) desaconseja su uso y lo restringe a situaciones puntuales [57], [58].
Inhibidores de JAK [*Jakinibis*]. Los inhibidores de JAK [*Janus Kinase*] son fármacos que bloquean la transmisión de la señal de activación de los receptores de numerosas citocinas (IL-6, IL-4, IL-17, IL-12, IL-10, etc.) y la consiguiente activación de los factores de transcripción STAT. Con ello se consigue una inhibición del efecto de la cascada de citocinas a diferentes niveles [59].
Anticuerpos monoclonales: Atendiendo al papel clave de las citocinas IL-1 y IL-6 en la cascada de inflamación, se han ensayado algunos anticuerpos que tratan de bloquear su efecto en los pacientes COVID-19:

Tocilizumab y sarilumab. El tocilizumab es un anticuerpo anti- receptor de la IL-6, que impide la unión de la IL-6 a su receptor, bloqueando así su acción sobre la célula diana. El sarilumab es un anticuerpo inhibidor de la IL-6. La AEMPS aconseja el uso de estos fármacos siempre que los niveles de IL-6 se encuentren por encima de 40 pg/ml [60], [58].

Anakinra. Se trata de una versión recombinante de un antagonista fisiológico del receptor de la IL-1 [61].

Otras aproximaciones que se barajan para el tratamiento de pacientes con COVID-19 son el uso de plasma hiperinmune de pacientes que hayan pasado la infección [62] o de anticuerpos monoclonales bloqueantes del virus [63]. Todos estos tratamientos se encuentran en fases de estudio y todavía carecen de una evidencia científica sólida [54].

Tal y como se ha comentado previamente, la utilización de diferentes terapias frente al COVID-19 se encuentra en continua revisión, y se publican periódicamente actualizaciones por la AEMPS [64].

## Búsqueda de la vacuna: pasado, presente y futuro

Desde la irrupción del SARS-CoV-2 se han puesto en marcha numerosas investigaciones y ensayos clínicos para desarrollar vacunas seguras y eficaces contra el virus [65].

Basándose en la experiencia de otros coronavirus como el SARS y el MERS, se están desarrollando posibles vacunas para SARS-CoV-2, dada su similitud estructural y genómica [66]. Las estrategias utilizadas incluyen el uso proteínas enteras del virus, péptidos virales, ARN mensajero (ARNm), o el propio virus inactivado.

Cabe destacar la necesidad de encontrar el antígeno adecuado capaz de generar una respuesta inmunitaria protectora, segura y duradera. En este sentido, la proteína S (espiga) del virus es la que ha demostrado, en estudios con SARS-CoV, tener una mayor capacidad de desarrollar tanto una respuesta humoral como celular efectivas [66]. La formación de anticuerpos neutralizantes frente a este antígeno implicaría el bloqueo de la unión del virus al receptor ACE2 y la consiguiente infección de la célula diana [67].

Las vacunas basadas en vectores virales ofrecen un alto nivel de expresión de proteínas y estabilidad a largo plazo, e inducen fuertes respuestas inmunes. El uso de adyuvantes también podría mejorar la inmunogenicidad [68] y prolongar la respuesta humoral [69]. También hay que tener en cuenta la generación de una respuesta inmunitaria celular de memoria para el desarrollo de estas vacunas [69]

Actualmente, existen más de 90 proyectos en marcha para la generación de una vacuna, algunos de ellos ya en fases clínicas iniciales [69], [70].En este momento, en el campo de la investigación y el desarrollo de tratamientos y vacunas contra el nuevo SARS-CoV-2 se están realizando grandes esfuerzos a nivel internacional. En los próximos meses podremos observar cómo evolucionan los distintos proyectos y en qué medida estas terapias se podrán aplicar a la población.
